# Slow CD4^+^ T-Cell Recovery in Human Immunodeficiency Virus/Hepatitis B Virus-Coinfected Patients Initiating Truvada-Based Combination Antiretroviral Therapy in Botswana

**DOI:** 10.1093/ofid/ofw140

**Published:** 2016-08-16

**Authors:** Motswedi Anderson, Simani Gaseitsiwe, Sikhulile Moyo, Kerapetse P. Thami, Terence Mohammed, Ditiro Setlhare, Theresa K. Sebunya, Eleanor A. Powell, Joseph Makhema, Jason T. Blackard, Richard Marlink, Max Essex, Rosemary M. Musonda

**Affiliations:** 1 Botswana Harvard AIDS Institute Partnership; 2 Department of Biological Sciences, University of Botswana, Gaborone; 3 Department of Immunology and Infectious Diseases, Harvard T. H. Chan School of Public Health, Boston, Massachusetts; 4 Division of Medical Virology, Faculty of Medicine and Health Sciences, University of Stellenbosch, Tygerberg, South Africa; 5 University of Cincinnati College of Medicine, Ohio

**Keywords:** Botswana, hepatitis B virus, HIV/HBV coinfection, tenofovir, Truvada

## Abstract

***Background.*** Hepatitis B virus (HBV) and human immunodeficiency virus (HIV) coinfection has emerged as an important cause of morbidity and mortality. We determined the response to Truvada-based first-line combination antiretroviral therapy (cART) in HIV/HBV-coinfected verus HIV-monoinfected patients in Botswana.

***Methods.*** Hepatitis B virus surface antigen (HBsAg), HBV e antigen (HBeAg), and HBV deoxyribonucleic acid (DNA) load were determined from baseline and follow-up visits in a longitudinal cART cohort of Truvada-based regimen. We assessed predictors of HBV serostatus and viral suppression (undetectable HBV DNA) using logistic regression techniques.

***Results.*** Of 300 participants, 28 were HBsAg positive, giving an HIV/HBV prevalence of 9.3% (95% confidence interval [CI], 6.3–13.2), and 5 of these, 17.9% (95% CI, 6.1–36.9), were HBeAg positive. There was a reduced CD4^+^ T-cell gain in HIV/HBV-coinfected compared with HIV-monoinfected patients. Hepatitis B virus surface antigen and HBeAg loss was 38% and 60%, respectively, at 24 months post-cART initiation. The HBV DNA suppression rates increased with time on cART from 54% to 75% in 6 and 24 months, respectively.

***Conclusions.*** Human immunodeficiency virus/HBV coinfection negatively affected immunologic recovery compared with HIV-1C monoinfection. Hepatitis B virus screening before cART initiation could help improve HBV/HIV treatment outcomes and help determine treatment options when there is a need to switch regimens.

Hepatitis B virus (HBV) and human immunodeficiency virus (HIV) coinfection represents a considerable health burden worldwide. As combination antiretroviral therapy (cART) has greatly improved survival in HIV-infected people, HBV has emerged as a major cause of morbidity and mortality in this group [1–3]. It is estimated that 5%–20% of the 35 million people living with HIV are also infected with HBV [4]. Sub-Saharan Africa has the highest burden of HIV/HBV coinfection [4]. In Botswana, HIV/HBV coinfection prevalence is 5.3%–10.6% [5, 6]. Human immunodeficiency virus/HBV coinfection worsens disease outcome more than having either infection alone. Human immunodeficiency virus has been shown to change the natural course of chronic HBV by increasing the likelihood of HBV infections acquired during adulthood to progress to chronic HBV infection [7], increasing rates of hepatitis e antigen positivity [8] and higher levels of HBV DNA [9] but lower alanine aminotransferase (ALT) levels and rapid liver disease progression [2]. Higher mortality has also been reported in HBV/HIV coinfection compared with HBV-monoinfected individuals [10, 11]. On the other hand, HBV has been reported to lead to higher baseline HIV viral load, lower CD4^+^ T cells, increased occurrences of advanced disease, lower body mass index (BMI), and increased mortality in HIV/HBV-coinfected participants compared with HIV-monoinfected individuals [12–15]. However, these data have not been replicated in other studies [16, 17].

The first-line HIV cART regimens may include drugs such as lamivudine, tenofovir, and emtricitabine that act against both HIV and HBV [18]. Screening for HBV before highly active antiretroviral therapy (HAART) initiation is standard of care in developed countries but not in resource-limited countries due to cost [15]. Hence, only HIV response is usually monitored in such settings. Most studies done on HBV/HIV coinfection are from low HBV-endemic areas, and these data may not be generalizable because the times of acquisition of HBV infection is in adulthood, whereas that of the high-endemicity areas is usually in early childhood [19]. Moreover, data from East Asia may not be applicable to Africa because of the different circulating genotypes and high HBeAg positivity in East Asia [19]. There are some studies that have been done to show the response of HBV to tenofovir, but most of them are not in Africa [17, 20, 21]. Most of the studies done in Africa were on response to lamivudine-containing regimens [1, 15, 22, 23]; however, starting from 2010, the World Health Organization recommended that the first-line regimen for HIV should contain tenofovir [24]. There is increasing access to tenofovir; thus, data on HBV response to tenofovir is important, especially in high HBV-endemic and resource-limited countries. There are conflicting data on the effects of HBV on HIV response to cART [1, 10, 11, 22, 23]. In this study, we determined HBV response to tenofovir containing first-line cART in HIV-infected individuals initiating cART by determining HBV surface antigen (HBsAg) loss, HBV e antigen (HBeAg) loss, and HBV deoxyribonucleic acid (DNA) levels at different time points during treatment. We also determined the effects of HBV coinfection on HIV response to cART.

## MATERIALS AND METHODS

### Study Participants

This was a retrospective longitudinal study. The research used archived plasma samples from HIV-infected adults who were initiating HAART. The samples were from a previous cohort—the Botswana National Evaluation Models of HIV Care (Bomolemo study): A Study Evaluating the Efficacy and Tolerability of Tenofovir and Emtricitabine (Truvada) as the Nucleoside Reverse Transcriptase Inhibitor (NRTI) backbone as first-line HAART for adults in Botswana—conducted by the Botswana Harvard AIDS Institute Partnership. This study enrolled 309 HIV-positive, treatment-naive adults who are 18 years or older and had CD4^+^ T-cell counts less than or equal to 250 or an acquired immune deficiency syndrome-defining illness. The participants signed an informed consent for enrollment into the study. The study was approved by the University of Botswana Institute Review Board and Human Research Development Committee at the Botswana Ministry of Health.

### Hepatitis B Virus Serologic Screening

The study screened 300 samples, which were available in storage (sample size was calculated based on the latest HBV/HIV prevalence in Botswana reported in literature [5, 25]). All samples were screened for HBsAg using the Murex HBsAg version 3 kit (Murex Biotech, Dartford, UK) according to the manufacturer's instructions. Samples positive for HBsAg were then screened for HBeAg using the Monolisa HBeAg-Ab PLUS kit (Bio-Rad, Hercules, CA). Both HBsAg and HBeAg losses were evaluated at 6, 12, 18, and 24 months. Hepatitis B core antibody (HBcAb) and hepatitis surface antibody (HBsAb) were evaluated at baseline using Monolisa Anti-HBC PLUS (Bio-Rad, Marnes-la-Coquette, Paris, France) and Monolisa Anti-HBS PLUS (Bio-Rad, Marnes-la-Coquette, Paris, France) enzyme-linked immunosorbent assay kits according to the manufacturer's instructions, respectively.

### Hepatitis B Virus Genotyping

The HBV genotypes were determined at baseline as previously described [26]. In brief, a 415-base pair fragment of the s gene was amplified in a seminested polymerase chain reaction using HBV 381 primer (5′-TGCGGCGTTTTATCATCTTCCT-3′; nucleotide [nt] 381–402) and HBV 840 primer (5′-GTTTAAATGTATACCCAAAGAC-3′; nt 840–861) for the first round [27]. The HBV 381 and HBV 801 primers (5′-CAGCGGCATAAAGGGACTCAAG-3′; nt 801–822) were used for second round [27]. The HBV genotypes and resistance mutations were determined using online databases (Stanford and Geno2Pheno) [28, 29] and confirmed by phylogenetic analysis. The evolutionary history was inferred by using the maximum likelihood method based on the general time-reversible model. Initial trees for the heuristic search were obtained automatically by applying neighbor-joining and BioNJ algorithms to a matrix of pairwise distances estimated using the maximum composite likelihood approach and then selecting the topology with superior log likelihood value. A discrete gamma distribution was used to model evolutionary rate differences among sites. The rate variation model allowed for some sites to be evolutionarily invariable. Evolutionary analyses were conducted in MEGA6.06 [30]. Sequences have previously been submitted to National Center for Biotechnology Information GenBank under accession numbers KR139680 to KR139749 [26].

### Quantification of Hepatitis B Virus Deoxyribonucleic Acid

Hepatitis B virus DNA levels were determined on samples that were HBsAg positive at baseline using COBAS AmpliPrep/COBAS TaqMan HBV Test, version 2.0 (Roche Diagnostics, Mannheim, Germany) with a limit of detection of 20 IU/mL. The HBV DNA levels were also determined at 6, 12, and 24 months posttreatment initiation. Hepatitis B virus DNA suppression was defined as undetectable HBV DNA. Virological breakthrough was defined as an increase of >1 log_10_ IU/mL in HBV DNA level from nadir in 2 consecutive measurements [31].

### Data Analysis

The relationship between HBV status and HIV ribonucleic acid (RNA) suppression (RNA <400 copies/mL), CD4^+^ T-cell count, liver enzymes (ALT and aspartate aminotransferase [AST]), BMI, and mortality were assessed using Wilcoxon rank-sum test and logistic techniques.

## RESULTS

### Characteristics of Participants

Approximately 64% of the 300 screened study participants were female, and the median age was 36 years (interquartile range [IQR], 32–42) (Table 1).

**Table 1.** Baseline Characteristics of Participants

Characteristic	HBsAg Positive (n = 28)	HBsAg Negative (n = 272^a^)	*P* Value
Age (IQR) years	35.5 (31.5–42.5)	36.0 (32–42)	.823
Female (N, %)	18.0 (64.3)	174.0 (64.0)	.575
BMI (IQR)	20.9 (18.9–23.6)	21.7 (19.1–25.1)	.686
CD4^+^ T-cell count (cells/mm^3^) (IQR)	171.6 (93.3–238.5)	166.3 (82.1–231.2)	.717
HIV viral load, log copies/mL (IQR)	4.9 (4.5–5.7)	5.1 (4.6–5.6)	.603
AST (IU/L) (IQR)	33.5 (24.4–43.9)	28.2 (23.0–36.4)	.148
ALT (IU/L) (IQR)	23.3 (16.0–36.7)	20.4 (14.8–28.8)	.172
Hemoglobin (g/dL) (IQR)	11.7 (9.6–13.2)	11.5 (9.9–12.9)	.981
FIB-4 score (IQR)	1.08 (0.8–1.5)	1.0 (0.8–1.4)	.355
Platelets (cells/μL) (IQR)	245.0 (201.5–300.0)	262.0 (208.0–316.0)	.462
NNRTI (N, %)			
Nevirapine-based regimen	15.0 (53.6)	146.0 (53.7)	
Efavirenz-based regimen	13.0 (46.4)	126.0 (46.3)	

Abbreviations: ALT, alanine aminotransferase; AST, aspartate aminotransferase; BMI, body mass index; FIB, fibrosis; HBsAg, hepatitis B virus surface antigen; HIV, human immunodeficiency virus; IQR, interquartile range; NNRTI, nonnucleoside reverse-transcriptase inhibitors.

^a^ One participant died.

### Hepatitis B Virus Serology Results

#### Hepatitis B Virus (HBV) Surface Antigen and HBV e Antigen Results

**Figure 1.**
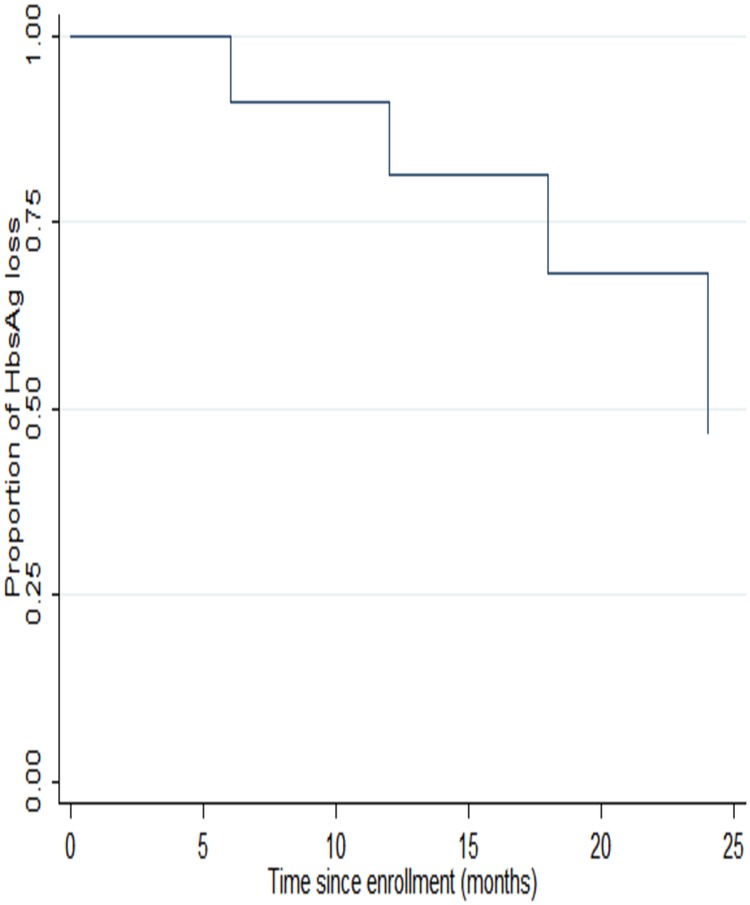
Kaplan–Meier curve for proportion of hepatitis B virus surface antigen (HBsAg) loss from baseline to 24 months.

Of the 300 participants, 28 were HBsAg positive, giving an HIV/HBV prevalence of 9.3% (95% CI, 6.3–13.2), and 5 of the 28 (17.9%; 95% CI, 6.1–36.9) were HBeAg positive. At 6 months, 10 of 27 (37%; 95% CI, 19.4–57.6) had lost HBsAg. The HBsAg loss at 12 and 18 months was observed in 9 of 25 participants (36%; 95% CI, 18%–57%). At 24 months, 9 of 24 participants (38%; 95% CI, 19%–59%) lost HBsAg. The median time to HBsAg loss was 168 days (IQR, 168–169 days) (Figure 1). Three participants lost HBsAg at 6 months but then became positive for all the subsequent visits. The other 2 participants lost the HBsAg at 12 months, but one of them had no available sample for testing at 24 months. In addition, there was 1 participant who was HBsAg negative at 24 months but also had no sample available for testing at 12 and 18 months. The CD4^+^ T-cell count did not affect HBsAg suppression at 6, 12, 18, and 24 months (*P* = .899, .167, .968, and .830, respectively). Of the HBeAg-positive individuals, 3 (60%) lost HBeAg by 6 months; the other 2 remained HBeAg positive for the duration of follow up. The HBeAg status did not affect baseline CD4^+^ T-cell count, AST, or ALT (*P* = .869, .348, and .224, respectively). CD4^+^ T-cell count did not affect HBeAg suppression at 6, 12, 18, and 24 months (*P* = .700, .906, .439, and .953, respectively). At 24 months postenrollment, there was no association between HBsAg loss and HBeAg loss (*P* = 1.000).

#### Hepatitis B Core Antibody (Ab) and Hepatitis Surface Ab Results

Hepatitis B core antibody was positive in 160 of the tested 295 participants (54.2%; 95% CI, 48.4%–60.0%), whereas 99 participants were positive for HBsAb (33.6%; 95% CI, 28.2%–39.3%). Of the 268 HBsAg-negative participants who were tested for HBV antibodies, 55 (20.5%; 95% CI, 15.9%–25.9%) were positive for HBcAb only, 12 (4.48%; 95% CI, 2.33%–7.69%) individuals were positive for HBsAb only, and 84 (31.3%; 95% CI, 25.8%–37.3%) were positive for both HBcAb and HBsAb (Table 2). The median age of participants who were HBsAb positive was 35.5 years (IQR, 31.5–46.5 years), whereas the median age of participants who were HBcAb positive was 38 years (IQR, 33–43 years). The ages were not significantly different (*P* = .746).

**Table 2.** HBcAb and HBsAb Results

Group	All (n = 295)	HIV (n = 268)	HIV/HBV (n = 27)
HBcAb only	73 (24.7)	55 (20.5%)	19 (70.4%)
HBsAb only	12 (4.1%)	12 (4.5%)	0 (0%)
HBcAb and HBsAb	87 (29.5%)	84 (31.3%)	3 (11.1)

Abbreviations: HBcAb, hepatitis B core antibody; HBsAb, hepatitis B surface antibody; HBV, hepatitis B virus; HIV, human immunodeficiency virus.

#### Hepatitis B Virus Deoxyribonucleic Acid Level Results

**Figure 2.**
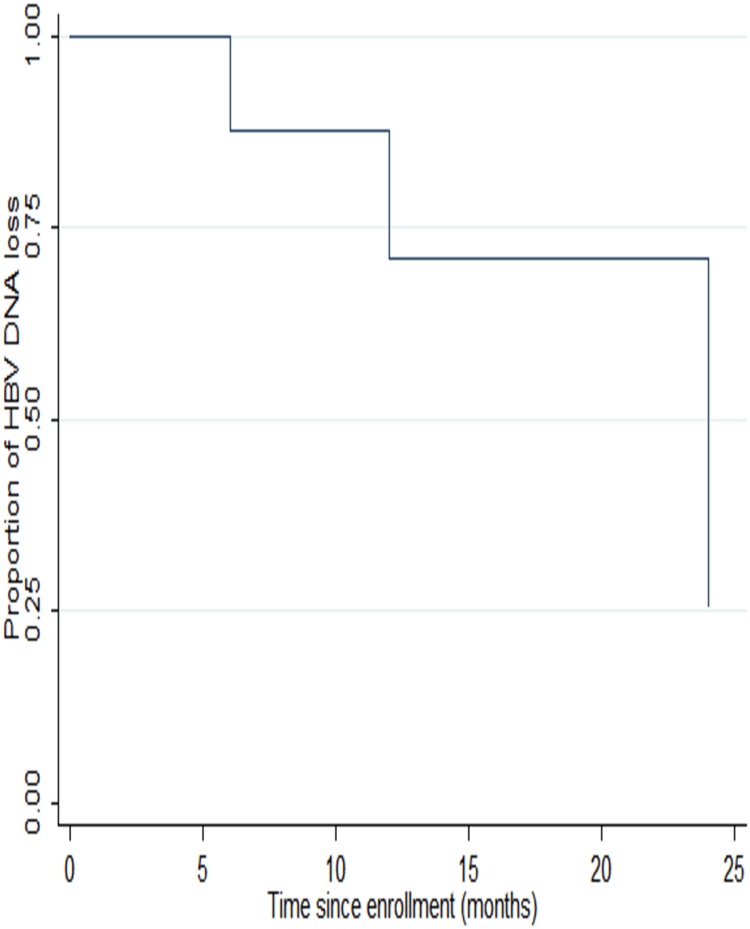
Kaplan–Meier curve for proportion of hepatitis B virus (HBV) deoxyribonucleic acid (DNA) loss from baseline to 24 months.

At baseline, 19 of the 28 HBsAg-positive (67.9%) samples had detectable HBV viral load with a median of 2500 IU/mL (IQR, 20–266 × 10^4^). The HBV viral load suppression increased with time on treatment—54% (95% CI, 33–74), 67% (95% CI, 45%–84%), and 75% (95% CI, 53%–90%) at 6, 12, and 24 months, respectively (Figure 2). The median time to HBV DNA loss was 336 days (IQR, 168–336 days). To further depict the rate of HBV DNA change over time, participants were categorized into 4 groups: target/DNA notdetectable (TND), HBV DNA <20 copies/mL, HBV DNA <10 000 copies/mL, and HBV DNA ≥10 000 copies/mL (Table 3). The number of participants in the TND group increased from 32% at baseline to 75% at 24 months postenrollment. The number of participants in the HBV DNA ≥10 000 copies/mL group decreased from 29% (baseline) to 0% (at 24 months postenrollment). All subjects who had loss of HBsAg and loss of HBeAg had HBV virologic suppression by 24 months. There was a significant association between CD4^+^ T-cell count and HBV viral load suppression at 12 months (*P* = .039) but not at 6 months (*P* = .535) and 24 months (*P* = .139). At 12 months, median CD4^+^ T-cell count was 373 cells/mL (IQR, 276–435) in participants who suppressed HBV viral load and 249 cells/mL (IQR, 202–308) in participants who did not suppress HBV viral load. At 24 months post-cART initiation, there was a significant association between HIV and HBV viral load suppression rate (odds ratio [OR] = 4.27; 95% CI, 1.69–10.74; *P* = .002). There was no significant association between HBV viral load at baseline and AST (*P* = .170) or ALT (*P* = .402). Participants who had baseline HBV viral loads >10 000 IU/mL were less likely to lose HBsAg, although this was not statistically significant (Table 4). Virological breakthrough did not occur in any participants. However, there was 1 participant who had HBV DNA <20 IU/mL at 12 and 24 months after having undetectable HBV DNA at 6 months. We could not classify this as virological breakthrough because we could not ascertain whether the true value of HBV DNA level was ≥10 IU/mL because it was below limit of detection of the assay.

**Table 3.** HBV DNA Suppression in the HIV/HBV Group

Time Since Enrollment	TND	HBV DNA <20 Copies/mL	HBV DNA <10 000 Copies/mL	HBV DNA ≥10 000 Copies/mL
Baseline	9 (32%)	5 (18%)	6 (21%)	8 (29%)
6 mo	14 (54%)	10 (38%)	2 (7%)	0
12 mo	16 (67%)	6 (25%)	2 (8%)	0
24 mo	18 (75%)	3 (13%)	3 (13%)	0

Abbreviations: DNA, deoxyribonucleic acid; HBV, hepatitis B virus; HIV, human immunodeficiency virus; TND, target/DNA not detectable.

**Table 4.** Baseline HBV Viral Load as a Predictor of HBsAg Loss

	HBsAg Loss Frequency	Odds Ratio (95% CI)	*P* Value
HBV viral load <10 000 IU/mL	70%	1	.131
HBV viral load ≥10 000 IU/mL	30%	0.242 (.0385–1.53)	

Abbreviations: CI, confidence interval; HBsAg, hepatitis B surface antigen; HBV, hepatitis B virus.

#### Hepatitis B Virus Genotypes

**Figure 3.**
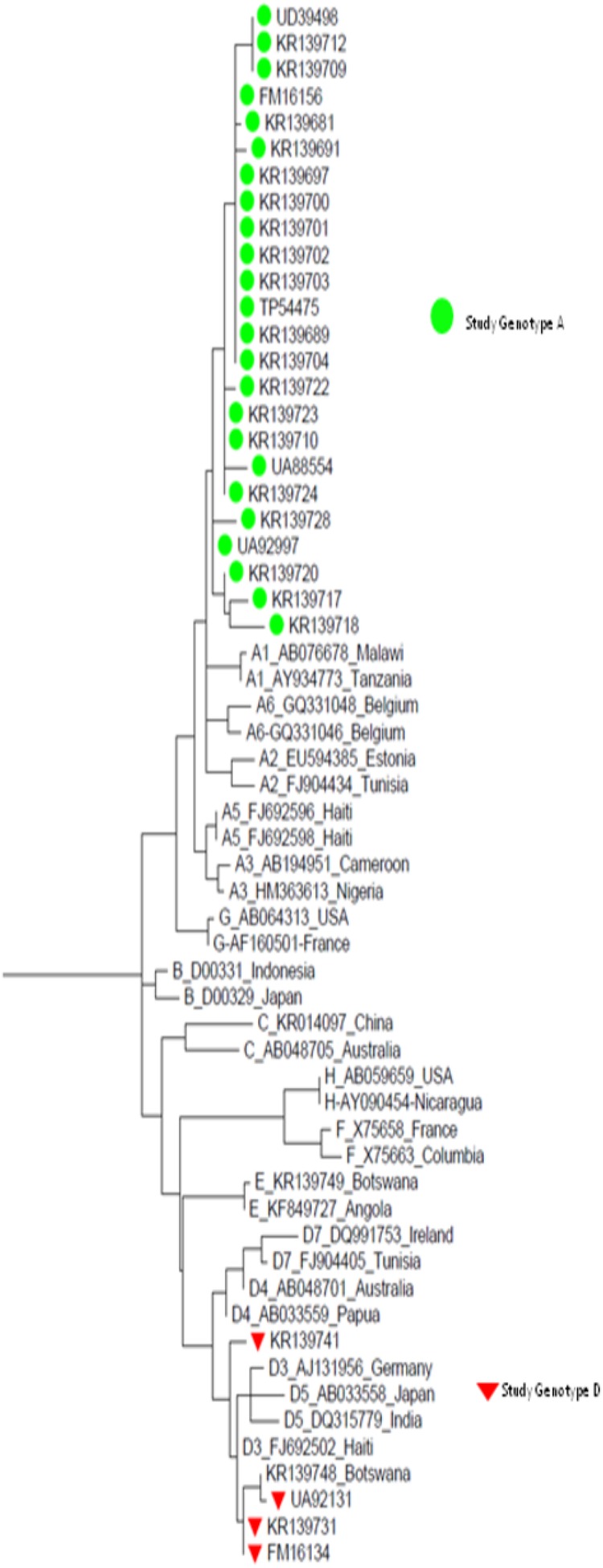
Maximum likelihood tree showing Botswana hepatitis B virus (HBV) fragment (415- base pair) and GenBank HBV references. Study isolates are marked by their accession and colored shapes (green for genotype A and red for genotype D), whereas the reference strains are represented by their subgenotypes, accession numbers, followed by their country of origin, as they appear in GenBank.

**Figure 4.**
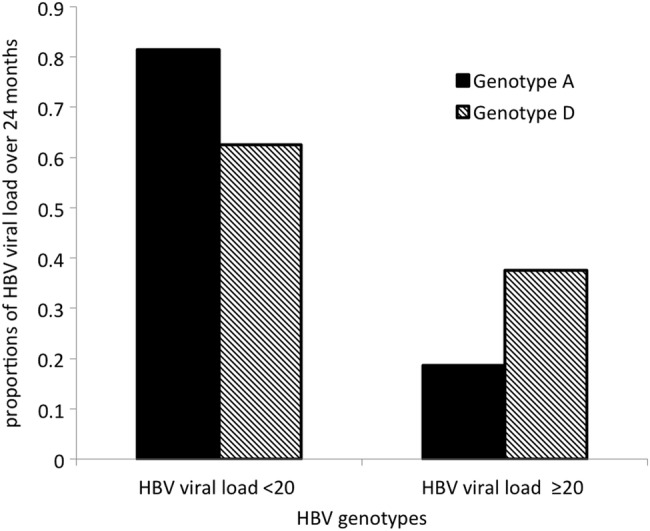
Comparison of hepatitis B virus (HBV) viral load proportions between HBV genotypes A and D after 24 months from enrollment.

The HBV genotypes were 24 (85.7%) genotype A and 4 (14.3%) genotype D (Figure 3). At baseline, no HBV resistance mutations were found. The median baseline CD4^+^ T-cell count in participants with HBV genotype A was 293 cells/mL (IQR, 216–397), whereas median baseline CD4^+^ T-cell count in participants with HBV genotype D was 274 cells/mL (IQR, 198–309). The results suggested no significant difference in CD4 T cells between the 2 genotype groups (*P* = .1366). There was an overall significant difference in CD4^+^ T-cell increase from baseline after 24 months between HBV genotypes A and D (*P* = .0019). Subjects with HBV genotype A had an overall average CD4^+^ T-cell increase of 144 cells/mL (95% CI, 120–169), whereas HBV genotype D subjects had an average CD4^+^ T-cell increase of 55 cells/mL (95% CI, 7–103). There was no siginificant difference in HIV viral load between HBV genotype A and D at baseline, 6, 12, 18, and 24 months (nor overall when time points were not considered). At 24 months, 90.0% (95% CI, 68.3–98.8) of the HBV genotype A subjects had HBV viral load <20 IU/mL, whereas 10.0% (95% CI, 12.3–31.7) had HBV viral load ≥20 IU/mL. Of the HBV genotype D subjects, 75.0% (95% CI, 19.4–99.3) had HBV viral load <20 IU/mL, whereas 25.0% (95% CI, .6–80.6) had HBV viral load ≥20 IU/mL (Figure 4). However, the difference in HBV viral load between HBV genotypes after 24 months did not reach statistical significance (Fisher's exact *P* = .437).

#### Human Immunodeficiency Virus-Related Outcomes

**Figure 5.**
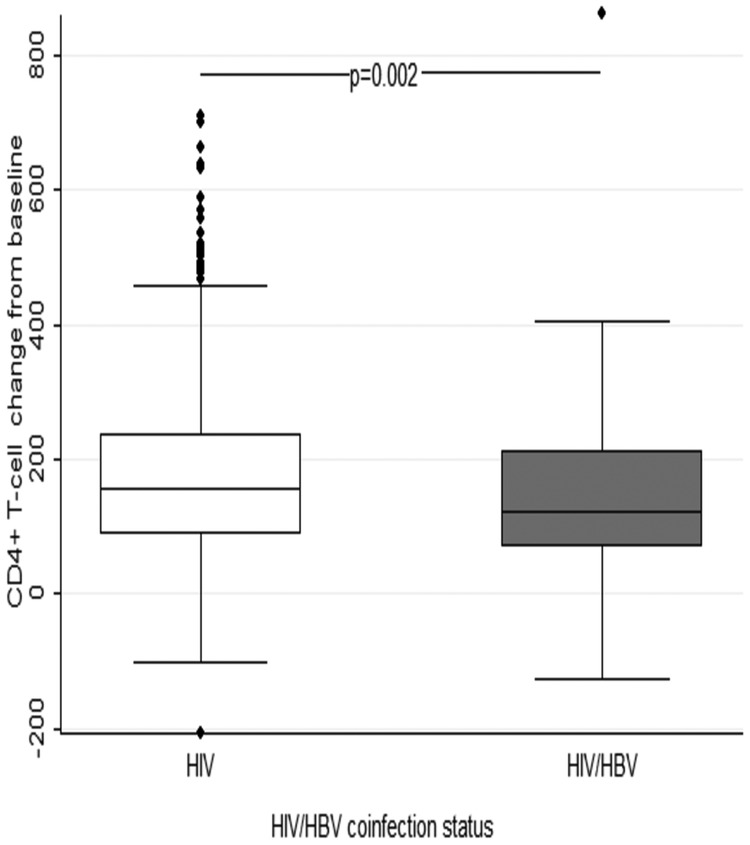
Comparison of CD4^+^ T-cell count change from baseline by hepatitis B virus (HBV) status to 24 months. Abbreviation: HIV, human immunodeficiency virus.

There was no significant differences by HBV status in sex (*P* = .575), BMI (*P* = .686), median CD4^+^ T-cell count (*P* = .880), AST (*P* = .172), or ALT (*P* = .148) at baseline. There were no observed statistically significant differences between the 2 groups in terms of ratios of mortality and hepatotoxicity (*P* = .603 and *P* = .132, respectively). After 24 months postenrollment, overall HIV viral load suppression in HIV-monoinfected group (73.7%; 95% CI, 71.1–76.4) was higher than the HIV/HBV-coinfected group (70.8%; 95% CI, 59.4–82.1); however, the difference was not statistically significant (*P* = .664). Overall, there was a higher CD4^+^ T-cell count increase from baseline to 24 months in HIV-monoinfected subjects (136 cells/mL; 95% CI, 129–143) compared with HIV/HBV-coinfected subjects (104 cells/mL; 95% CI, 75–132; *P* = .002) (Figure 5). However, HBV status was not a predictor of CD4^+^ T-cell gain at 12 months from enrollment (adjusted OR = 0.72; 95% CI, .20–2.65) (Table 5).

**Table 5.** Predictors of CD4^+^ T-Cell Gain at 12 Months

	Univariate		Multivariate^a^	
Variable	Odds Ratio (95% CI)	*P* Value	Odds Ratio (95% CI)	*P* Value
Age	0.86 (0.44–1.68)	.668	0.92 (0.47–1.82)	.810
HBV status	0.72 (0.20–2.65)	.624	0.67 (0.20–2.79)	.672
HBV vl suppression	2.33 (0.26–20.7)	.446	1.34 (0.11–16.4)	.819

Abbreviations: CI, confidence interval; HBV, hepatitis B virus; vl, viral load.

^a^ Twelve months adjusting for age and sex.

**Figure 6.**
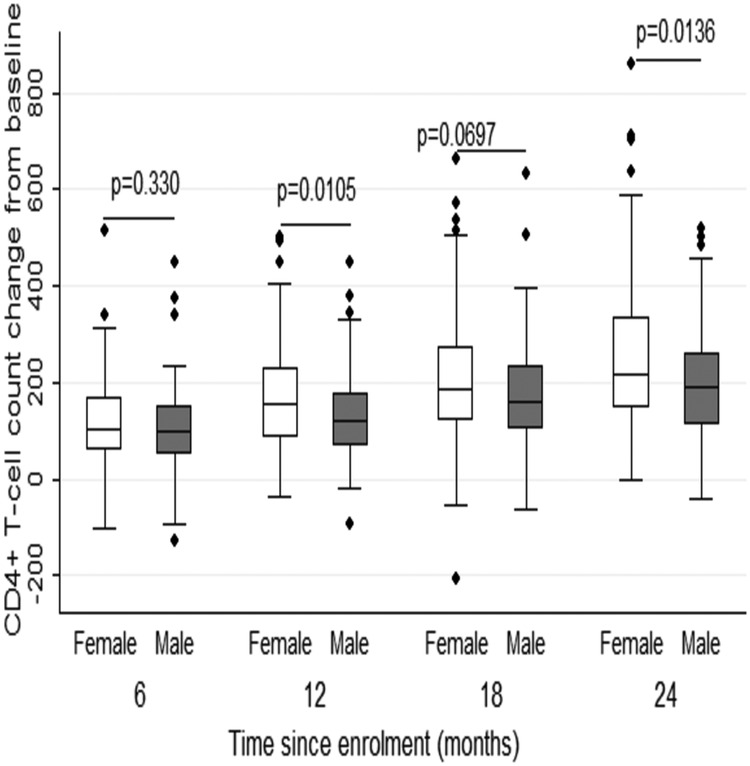
Comparison of CD4^+^ T-cell count change from baseline by sex.

When comparing CD4^+^ T-cell gain by sex, females gained more CD4^+^ T cells at 12 months and 24 months than their male counterparts (Figure 6). Furthermore, overall CD4^+^ T-cell gains significantly differed by sex (*P* = .001). There was no significant difference in CD4^+^ T-cell gains by age at 6, 12, 18, and 24 months (data not shown). Furthermore, in a univariate analysis of predictors of CD4^+^ T-cell gains, HBV status, HBV DNA suppression, and age were not significantly associated with CD4^+^ T-cell gains (Table 4). In the multivariate analysis of CD4^+^ T-cell gains adjusting for age and sex, there was no significant association of HBV status, HBV DNA suppression, and age as predictors of CD4^+^ T-cell gains. Eighty-five percent of the cases that had suppressed HIV viral load had gained 157 (Q1, Q3: 107–230) CD4^+^ T cells at 12 months.

## DISCUSSION

In this study, we report for the first time the impact of Truvada-based cART on HIV/HBV-coinfected patients compared with HIV-1-monoinfected patients in Botswana. Overall, both coinfected and HIV-1-monoinfected patients responded well to the Truvada-based cART, with HIV-1 infection markers CD4^+^ T-cell counts and HIV-1 viral load showing positive response. Likewise, HBV infection markers HBsAg and HBeAg were lost in some of the participants. Furthermore, HBV viral load was also suppressed in most participants. However, the HIV/HBV-coinfected patients had a slower CD4^+^ T-cell gain compared with the HIV-1-monoinfected patients.

The HIV/HBV prevalence is comparable to previous reports within the region, which range from 4.2%–22.9% in Zimbabwe and South Africa [6, 32–34]. We also found similar rates of HBeAg, HBcAb, and HBsAb as reported previously in Botswana [6].

The HBsAg loss documented in this study (38%) was higher than what has been reported in other studies [35–37]. The high and rapid HBsAg loss might be partly due to genotypes A and D, which have been associated with a rapid initial HBsAg reduction that correlates with HBsAg loss [38]. The study conducted in South Africa and Zambia also had a higher HBsAg loss (18%) within 12 months [35] compared to studies such as those in the Ivory Coast, where HBsAg loss was as low as 6.2% after a median of 12.3 months on treatment [39], but it was lower than this cohort. The difference might be the differences in genotypes because 22% of the Hamers study were genotype E [35]. However, the Hamers study only checked for HBsAg loss at 12 months and not at 6 months [35]. Furthermore, Zoutendijk et al [37] noted that most of HBsAg loss happens during the first year [40], which might explain the rapid HBsAg loss in our study. On the other hand, the HBV DNA suppression is comparable to other studies [35, 41]. There are other studies that have also demonstrated that HBV virologic suppression increased with time on treatment [42]. Baseline CD4^+^ T cells were significantly associated with HBV DNA suppression at 12 months but not at other visits. This concurs with other studies such as Kim et al [43] who reported baseline CD4^+^ T cells as a predictor of HBV suppression in response to tenofovir [44].

The pretreatment CD4^+^ T-cell counts in this study were similar between the 2 groups as reported by Kim et al [43] in Kenya [10]. This was also similar to another study that included Botswana and South African pregnant women, and the baseline CD4^+^ T cells for Batswana were similar between the HIV/HBV-coinfected and HIV-monoinfected group, whereas that of South Africans were lower in the HIV/HBV-coinfected group [45]. Other studies in South Africa and Tanzania reported marginally lower baseline CD4^+^ T cells [17, 35]. Some studies found similar CD4^+^ T-cell response in both groups [1, 15, 23, 35]. However, we documented a lower CD4^+^ T-cell response in HIV/HBV-coinfected participants compared with HIV-monoinfected participants, similar to the results found in the large cohort from Tanzania [17]. This large cohort from Tanzania also reported a marginally significant recovery at 12 months; however, in our cohort, there were no significant differences in immunologic recovery between HIV/HBV-coinfected and HIV-monoinfected participants at 12 months [17]. The differences might be due to the smaller number of the participants in our cohort. Furthermore, another study in Southern Africa also reported no difference in CD4^+^ T-cell recovery at 12 months after cART initiation [35]. The lower CD4^+^ T-cell response in HIV/HBV-coinfected could be due to destruction of CD4^+^ T cells by HBV-mediated T-cell activation [46].

Comparable hepatotoxicity between HIV/HBV-coinfected and HIV-monoinfected participants was observed, as reported by others [16, 47–49]. However, some studies in South Africa, Ghana, Kenya,Tanzania, and Cambodia have shown increased hepatotoxicity in HIV/HBV-coinfected participants [1, 11, 17, 22, 23].

Pretreatment HIV-1 RNA was similar in both HIV/HBV-coinfected and HIV-monoinfected participants, as reported in other studies [1, 15, 22]. On the other hand, higher baseline HIV RNA in HIV/HBV-coinfected participants has been documented [13]. In this study, similar to studies conducted in South Africa and Kenya, there was no difference in HIV viral load suppression between the 2 groups [1, 15, 22]. This is contrary to a study conducted in Taiwan, which reported a lower HIV viral load suppression in HIV/HBV-coinfected participants compared with the HIV-monoinfected group [50].

The HBV genotypes found in this cohort are consistent with what have been reported in the country [26] and within the region [51]. There was no significant difference in HBV DNA level at all time points between genotype A and D. The lack of association between HBV genotype and HBV DNA load has been reported before [12, 52], but in our cohort it might be due to small numbers. However, some studies have recorded differences between genotypes, and genotype D was found to have a higher HBV viral load [53]. Genotype D participants had lower CD4^+^ T-cell responses than participants with genotype A. Genotype D has been associated with a worse outcome in some studies [54, 55], which might explain the worse immunosuppression in these individuals.

## LIMITATIONS

The limitations of this study include small sample size. The HIV/HBV prevalence might be underestimated, because it does not include occult HBV. There is a need in future to generate data in a larger sample size that can be extrapolated to the general population. Furthermore, the cohort was predominately female, hence data may not be generalizable to men. Another limitation of this study is that HBcAb immunoglobulin M screening, a test for hepatitis acute infection, was not performed; however, most of the infections in sub-Saharan Africa are acquired during childhood, and this was a cohort of adults. Hence, we could assume that most of the infections were chronic infections. The clinical significance of unsuppressed HBV DNA need to be studied further in this setting.

## CONCLUSIONS

In conclusion, HIV/HBV coinfection might have a negative effect on the immunologic response of HIV-1 to cART; hence, screening and monitoring of HIV/HBV-coinfected participants might be important in this setting. Most participants suppressed HBV DNA, and the HBV serologic response was higher than in most studies. This supports the recommendation of Truvada-based cART in HIV/HBV coinfection. However, because approximately 25% of the participants did not completely suppress HBV DNA after the 2-year follow up, it is crucial to monitor HBV response in coinfected patients on Truvada-based cART so that in case of treatment switch for HIV virologic failure, the patients are not put on a regimen that is less effective against HBV and could result in the rebound of HBV viremia.
